# Developmental biology is poised to discover altogether new principles in biology in the 21st century

**DOI:** 10.1016/j.ydbio.2022.05.004

**Published:** 2022-05-14

**Authors:** Alejandro Sánchez Alvarado

**Affiliations:** Stowers Institute for Medical Research, Howard Hughes Medical Institute, Kansas City, MO, United States

## Abstract

In the 20th century, developmental biology spearheaded a revolution in our understanding of complex biological problems. Its success rests in great part on a truly unique approach that has recruited a diversity of systems and research organisms rather than focusing on isolated cells or molecules, while also employing a wide variety of technological and intellectual approaches. But what will developmental biology contribute to this century? Advances in technology and instrumentation are presently moving at neck-breaking speed and herald the advent of an age of technological wonders in which previously inaccessible biology is now tangibly within our grasps. For instance, single-cell RNAseq has revealed novel, transient cell states in both stem and differentiated cells that are specified by defined changes in gene expression frequency during regeneration. Additionally, genome-wide epigenetic analyses combined with gene editing and transgenic methodologies have identified the existence of regeneration responsive enhancers in adult vertebrate tissues. These circumstances combined with our discipline’s diversity of experimental and intellectual approaches offer unimaginable opportunities for developmental biologists not only to discover new biology but also to reveal entirely new principles of biology.

“More than once, haste and a predilection for the fantastic have led naturalists into error and have concealed from them what they otherwise could have recognized easily. It is not enough to say, therefore, that one has seen such and such a thing. This amounts to saying nothing unless at the same time the observer indicates how it was seen, and unless he puts his readers in a position to evaluate the manner in which the reported facts were observed.”

Abraham Trembley 1744 Memoirs (Lenhoff and Lenhoff, 1986).

“The historical questions with which so many problems seem to be connected, and for which there is no rigorous experimental test, are perhaps responsible for the loose way in which many problems in biology are treated, where fancy too often supplies the place of demonstration.”

T.H. Morgan (1901), Regeneration (Morgan, 1901).

Over a century and a half separate the above quotes from two superb experimentalists writing about regeneration in their respective book introductions. That both authors had to decry a tendency for self-deception in biology underscores why, when compared to other sciences, biology may have taken such a long time to find its footing. Indeed, it was not until the 20th Century that practitioners of developmental biology began to unravel some of life’s most complex and mysterious processes. They discovered morphogens, genetically defined and mechanistically dissected the major signaling pathways by which cells communicate with each other. They also uncovered fundamental principles of differential gene regulation, and along the way discovered and characterized the fundamental underpinnings of embryogenesis and pattern formation (recognized by a Nobel Prize in 1995 to Drs. Nüsslein-Volhard and Weischaus), gastrulation, organogenesis, neurogenesis and sex determination, cell and tissue polarity, epigenetics, microRNAs, aging, cell death (recognized by another Nobel Prize in 2002 to Brenner, Horvitz and Sulston), and cellular reprograming (also recognized by a Nobel Prize in 2012 to Gurdon and Yamanaka).

It is easy to forget, yet important to remember, that it was developmental biologists who first isolated and cultured stem cells, cloned animals, and provided important technological advances ranging from *in situ* hybridization, to genome manipulation by homologous recombination (Nobel Prize in 2007 to Capecchi, Evans and Smithies), to *in vivo* imaging, and RNA-mediated genetic interference or RNAi (also recognized by a Nobel Prize to Andy Fire and Craig Mello in 2006). As such, developmental biology in the 20th century provided the foundations for stem cell biology and tissue engineering and crafted the context in which to understand human birth defects and disease (Dunwoodie and Wallingford, 2020).

The accomplishments of our field thus far have been numerous. And today, work in developmental biology has started to provide a rich playing field for a broad swath of biological sciences. And yet, throughout my career, I have heard many argue that the best years of developmental biology are well behind us, never to come back. In fact, such end-of-era concerns have been a recurring theme in our field for decades. Despite such decades-long negative assessments and forecasts, developmental biology continues to flourish and today occupies the center stage in the life sciences (Wallingford, 2019). Such success is likely a consequence of the field studying a diverse array of systems and research organisms rather than isolated cells or molecules using a wide variety of technological and intellectual approaches. As studies in developmental biology continue to forge ahead and expand into new areas such as regeneration, epigenetics, stem cells, genomics, systems biology, and growth control, we can expect major contributions in the coming years. In fact, I predict that developmental biology is likely to lead us to the discovery of entirely new principles in biology, particularly as the field expands into the study of post-embryonic, post-natal, and adult biology. In this short essay, I will attempt to illustrate this point using adult animal regeneration (Fig. 1) as a paradigm to inform not only development but also fundamental aspects of animal biology, including our own.

## 1. Regeneration: developmental biology’s wild frontier

After an exhaustive study of animal regeneration, T. H. Morgan once wrote: “The fact that the process of regeneration is useful to the organism cannot be made to account for its existence in the organism” (Morgan, 1901). Examples abound to support his conclusion. The nemertine worm *Lineus ruber* and its close relative *Lineus viridis* provide a great case in point. These two species are almost identical in morphological attributes and share similar environmental niches competing for the same resources. And yet, they respond very differently to amputation. Amputate a part of *L. ruber* and the missing part regenerates; amputate a part of *L. viridis* and no regeneration is observed (Brockes et al., 2001). Such intraphyletic variability is not uncommon (Needham, 1952), and can readily be extended across multiple phyla (Sánchez Alvarado, 2000) where regeneration is found broadly yet unevenly distributed. Why, then, can some animals regenerate missing body parts and others cannot? We are currently at odds in explaining regeneration as an evolutionary variable (Bely and Nyberg, 2010; Brockes et al., 2001; Maden, 2018), and still lack sufficient molecular evidence to resolve whether regeneration is a primordial metazoan attribute or has evolved independently multiple times during animal evolution.

Regeneration provides us with a rich tapestry of problems that are becoming more and more vulnerable to experimentation. For instance, systematic studies of how the mechanisms of regeneration formally compare to planarian embryogenesis are now possible (Davies et al., 2017). Such efforts help address the long-standing question of whether regeneration is simply a recapitulation of development or the result of independent mechanistic innovations. How, for instance, are embryonic stem cells functionally different from adult stem cells? Are the same genetic choreographies of gene expression and translation used during embryogenesis to organize the body axes and facilitate organogenesis the same or different during regeneration? These studies may ultimately help resolve a particularly vexing paradox. Why are organisms that display an impressive ability to undergo regulative embryonic development after injury such as the mouse, fruit fly, and frog are such poor adult regenerators? Understanding the molecular and cellular basis of this discrepancy may help us identify the central differences crucial to preserving regenerative abilities into adulthood. In fact, a host of questions remain unanswered. Among these are how do tissues regulate allometric growth. In other words, how are the scale and proportion of a regenerating body part regulated so they can reach their original size? And how do newly regenerated body parts like a hand (autopod) or a head, for that matter, functionally integrate with pre-existing tissues?

## 2. Let animals tell us their story in their own “words”

In the first decades of the 20th century, developmental biology relied on a theoretical framework that was based mostly on observation and limited yet fiendishly clever experimental perturbations. To help communicate ideas and findings across different species and biological contexts, the field had at its disposal an extensive list of terminology. It is important to remember, however, that the vast majority of these terms were coined in the early 1800’s, a time where science knew little to nothing about genetics, epigenetics, evolution, and cell biology, for example. Terms like gastrulation, epiboly, holoblastic and meroblastic cleavage, animal and vegetal poles, micromeres and macromeres, blastopore, archenteron, blastocoel, germ layer, mesoderm, ectoderm, endoderm, deuterostome, protostome, triploblastic and diploblastic, etc. Terms that are all in use today and seem to have made it to the 21st century mostly unquestioned and unscathed.

While the above terminology has been useful, we possess today a remarkably sophisticated and growing technological armamentarium to interrogate biology, technology that can see farther and with higher resolution in time and space than ever before in the history of science. A truly unprecedented state-of-affairs that makes previously inaccessible biology amenable to observation and experimentation. Thus, it is reasonable to ask whether attempting to explain and frame new biology within old and possibly unsuitable terminology, we should instead allow the actual genetic, epigenetic, biochemical, genomic, and cellular data tell us the story of development. All of these processes can now be completely and rigorously measured not only in parallel, but also exponentially.

The continuous miniaturization and increasingly smaller sample sizes required to study complex biology is a consequence of the development of exponential technologies that allow us to parallelize processes aimed at measuring global changes in a system. Among these are microfluidics, artificial intelligence, machine learning, neural networks, and advances in optical and electron microscopy. Nowhere has the effect of exponential technologies become more evident than in molecular biology, as demonstrated by the evolution of methods used to sequence nucleic acids from Sanger and Maxam and Gilbert (Maxam and Gilbert, 1977; Sanger et al., 1977) to current next generation sequencing methods (Gkazi, 2021). It is, therefore, reasonable to argue that going forward those disciplines adopting exponential technologies stand the best chance to expose completely new vistas of biology. As these technologies begin to spill over into the worlds of amino acids, lipids, and carbohydrates, for example, we should anticipate that the secret life and dynamics of proteins and other biomolecules may finally be revealed in all their dimensions, from rate of turnover to the structural states occupied at particular moments of their functions. Consider Alpha Fold, for example (Avsec et al., 2021; Jumper et al., 2021a, 2021b; Tunyasuvunakool et al., 2021). This novel method—based on an exponential computational method involving neural networks and machine learning—incorporates physical and biological knowledge about protein structure, as well as multi-sequence alignments to design a deep-learning algorithm that in the majority of cases tested accurately competed with experimental structures and handily outperformed other methods of protein structure prediction (Jumper et al., 2021a). And this, of course, is just the beginning.

The gamut of biological processes exponential technologies is making available to interrogation share a common denominator: the passage of time. And time is an essential component of development. Hence, we should expect that if new principles of biology are to emerge in this century, they are likely to be first detected and measured in the study of developmental processes, particularly in the growing list of species that such technologies are now making accessible and thus vulnerable to experimentation. And regeneration, currently exploring diverse animals such as planarians, hydra, nematostella, zebrafish, killifish, medaka, axolotls, salamanders, echinoderms, hemichordates and urochordates, among many others, stands to contribute significantly to our collective effort to unravel the secrets of life.

## 3. The temporal transformation of tissues: plasticity

For regeneration to manifest itself, tissues responding to injury must engage in activities that are not necessarily demanded of them during their normal physiological functions. Thus, the response of pre-existing differentiated cells may determine whether an appendage, for example, can or cannot be regenerated after amputation, for example. Even though regeneration has been the subject of extensive phylogenetic, developmental, cellular, and molecular studies, the mechanisms underlying the broad disparity of regenerative capacities in animals remain elusive. Therefore, the ability and inability of animals to regenerate tissues lost to amputation provide us with an opportunity to study cell and tissue plasticity.

It has been shown in planarians that postmitotic, differentiated tissues in amputated fragments that are devoid of stem cells can reprogram their genomic output and both express (Reddien et al., 2007) and repress (Gurley et al., 2010) patterning signals. Such modulation is necessary for the amputated fragments to dramatically rearrange pre-existing tissues to produce small animals and restore anatomical forms and functions of appropriate allometric proportions. These observations suggested that reprograming mechanisms in differentiated cells must exist that allow rapid spatial and temporal changes of expression of signaling proteins regulating planarian body patterning.

Similar plasticity is also known to occur in vertebrates such as the teleost fishes, where expression of signaling pathways like Wnt (Stoick-Cooper et al., 2007) and FGF (Hyde et al., 2012) are reactivated during the regeneration of caudal fins. Because changes in cis-regulatory elements have been shown to be a major source of morphological diversity (Long et al., 2016; Rebeiz et al., 2011; Vierstra et al., 2014; Yue et al., 2014), enhancers responding to injury have been postulated to explain the adult re-expression of genes associated with signaling pathways best characterized during embryogenesis. In fact, evidence has emerged demonstrating the existence of injury-responsive enhancer elements. However, ablations of these characterized elements in zebrafish (Kang et al., 2016) and fruit flies (Harris et al., 2016) have shown that they are generally dispensable for regeneration. Therefore, whether conserved regeneration-responsive, rather than injury-responsive, elements exist in vertebrate genomes and how they evolved has remained an open question.

However, recent studies in the African killifish have provided evidence for the presence of elements in the vertebrate genome that may allow differentiated cells to launch a regenerative response after injury, and that such elements may differ between regeneration competent and incompetent organisms (Wang et al., 2020). In this study a comparison of the regeneration response in two closely related fish species the zebrafish *Danio rerio* and the African Turquoise killifish *Nothobranchius furzeri* uncovered large differences in the genomic response of these species to amputation. Importantly, it also revealed an evolutionarily conserved subset of responses which together are referred to as a regeneration response program (RRP). This response included known effectors of regeneration such as FGF and Inhibin, both of which are differentially activated in rodents with robust (*Acomys cahirinus*) and weak (*Mus musculus*) regeneration responses (Brant et al., 2019; Gawriluk et al., 2016; Wang et al., 2020). A closer inspection of the genes encompassed by the RRP, revealed that they most were accompanied by upstream regions of DNA enriched in H3K27ac and H3K4me3 marks that suggested the presence of putative enhancers. Functional testing of these regions of DNA via systematic transgenesis analyses demonstrated their function as regeneration-responsive enhancers (RREs). Interestingly, the identified enhancers required the presence of activator protein 1 (AP-1) binding motifs, suggesting a role in regeneration for this ancient regulator of gene expression (Toone and Jones, 1999).

**Fig. 2.**
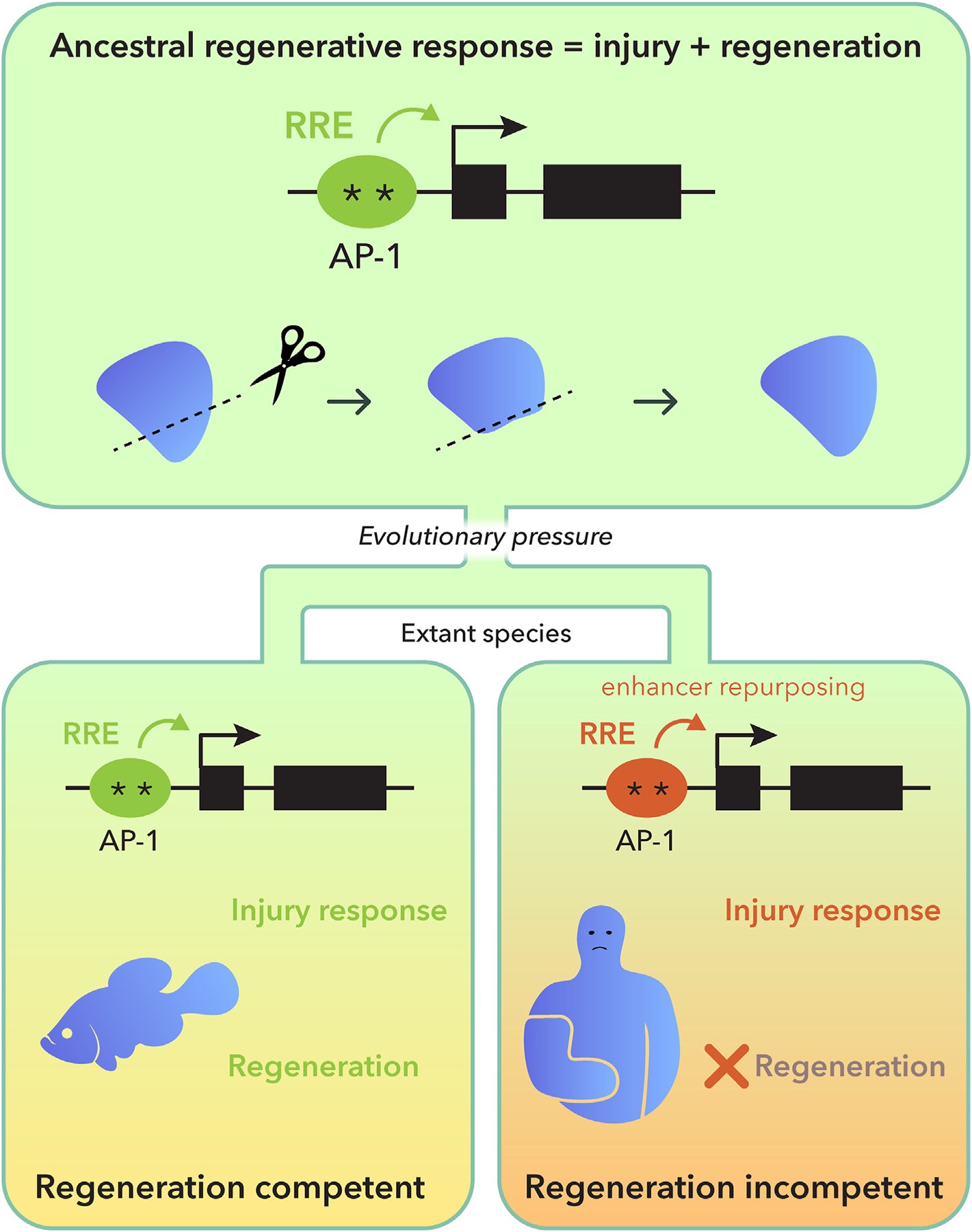
Regeneration responsive enhancer model for loss of regenerative capacities during evolution. We postulate that the ancestral function of AP-1 motif–enriched enhancers was to activate a regenerative response, and that through the course of evolution and speciation, regeneration and injury responses became dissociated from each other in some, but not all, enhancers. In extant species, regeneration-competent animals maintain the ancestral enhancer activities to activate both injury response and regeneration, whereas repurposing of ancestral enhancers in regeneration-incompetent animals led to loss of regenerative capacities. Repurposing of ancestral regulatory sequences to generate new regulatory functions is not without precedent and has been well documented in both vertebrates and invertebrates (Cretekos et al., 2008; Frankel et al., 2011; Vierstra et al., 2014). Our model provides a testable hypothesis to explain the uneven distribution of regenerative capacities in vertebrates. Modified from (Wang et al., 2020).

Based on these results, we proposed an RRE-based model for the loss of regenerative capacities in animals during evolution (Wang et al., 2020). In this model, the ancestral function for AP-1–enriched RREs is to activate a regenerative response that included both injury and regeneration. Through the course of evolution and speciation, regeneration and injury responses may have become dissociated from each other in some but not all enhancers. In extant species, regeneration competent animals maintain the ancestral enhancer activities to activate both injury and regeneration responses, whereas in regeneration incompetent animals, repurposing of the ancestral enhancers may have led to the retention of injury response activities but to the loss of the regeneration response (Fig. 2). Identifying the components of AP-1 and characterizing their functions may provide a mechanistic understanding of how cells can modify their genomic output to generate the tissue plasticity required in adult animal tissues to launch an effective regenerative response to injury.

## 4. Cell types versus cell states

**Fig. 3.**
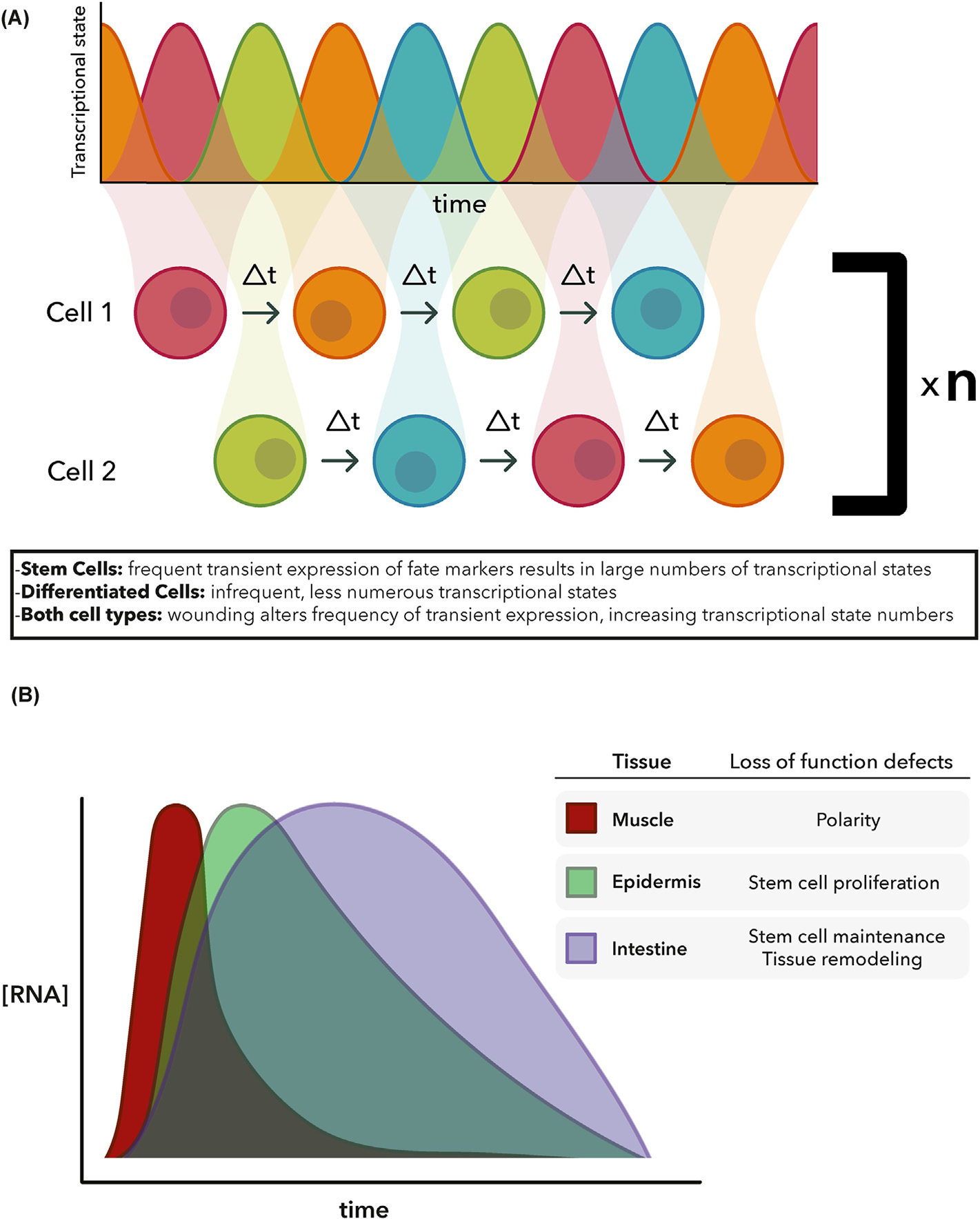
Models of cell state dynamics in stem and differentiated cells. (**A**) Probabilistic model in which “n” numbers of stem and differentiated cells transiently express different gene profiles (RNA concentration represented by curves, with colors representing different genes being expressed at different times by a cell). Wounding is proposed to alter the frequency and/or persistence of expression of gene profiles, thus producing the necessary cell types required for regeneration. Modified from (Adler and Sánchez Alvarado, 2015) (**B**) General dynamics of TRACS during regeneration for muscle, epidermal, and gut cells. Shown are the general effects on specific aspects of regeneration that were perturbed by genetic disruption of genes associated with each cell state (Benham-Pyle et al., 2021).

Earlier studies of planarian regeneration and neural crest cells revealed an unexpected degree of non-genetic heterogeneity in individual cells of seemingly homogeneous cell populations (Eisenhoffer et al., 2008; Huang, 2009). Such findings open the door to considering how dynamic processes may maintain particular states for defined periods of time and how such states may be exited during development or in response to changes of tissue homeostasis triggered by environmental damages such as injury and amputation. In an effort to incorporate these new findings to the study of regeneration, we posited a non-standard model for stem cells, in which their properties during homeostasis and regeneration could be explained probabilistically (Adler and Sánchez Alvarado, 2015). In this model, self-renewal becomes a conceptual property not permanently possessed by a discrete population, but transiently held by a small number of cells and arising probabilistically depending on the demands of the animal (Adler and Sánchez Alvarado, 2015). If these stem cells stochastically express progenitor markers for specific organs, perhaps injury induces changes in the frequency or periodicity of expression, resulting in altered differentiation of stem cell progeny. Such a model allows us to frame the remarkable plasticity of planarian in terms of dynamic cell states rather than statically defined cell types (Fig. 3A).

The advent of single-cell sequencing has confirmed not only that cell types are underpinned by transcriptional heterogeneity, but also that such heterogeneity is more likely to be the rule rather than the exception (Morris, 2019). In planarians, we and others demonstrated that the stem cells of this organism, which are characterized by the expression of a member of the *argonaute* family of genes known as *piwi*, are composed of cells with diverse expression profiles (van Wolfswinkel et al., 2014; Zeng et al., 2018). Purification of neoblasts expressing high levels of *piwi* and subjected to single-cell RNA sequencing were shown to exist in at least 12 different clusters, suggesting the existence of many subpopulations and cell states that can be populated by neoblasts. Additionally, one of this cell clusters (NB2) was shown to modulate its expression profile depending on whether the cells were in intact, partially irradiated or regenerating tissue, indicating that cell clusters can occupy diverse cell states as tissues undergo temporal transformations (Zeng et al., 2018). In retrospect, our ability to identify these dynamic states of a cell’s life history were likely limited by the methods of study being employed: fixed analysis of tissues and single-timepoint analysis of transcriptomes. With technologies that permit the observation of cell behavior and transcriptional output over time, it has become increasingly clear that cell types are not static, and though anatomical position and cell function may be fixed, stochastic and transient changes are likely and incessantly occurring at the cellular level.

## 5. Stable versus terminal differentiation

If “terminally differentiated” tissues can change gene expression and change their functions in response to environmental changes and insults, are they truly “terminally differentiated” or just stably differentiated? (Sánchez Alvarado and Yamanaka, 2014). Historically, the function of differentiated cells during regeneration has been largely dismissed as secondary to the action of stem cells. The role of differentiated cells has been appreciated mostly within the context of a cellular microenvironment known as a niche, which protects and maintains stem cells (Morrison and Spradling, 2008). Considering that planarian regeneration involves both local restoration of missing tissues (Sánchez Alvarado and Newmark, 1999), and a simultaneous reproportioning of the entire body plan (Reddien and Sánchez Alvarado, 2004), one cannot avoid suspecting that differentiated cells likely play important roles in regeneration on scales larger than what has been previously described for a stem cell niche (Elliot and Sánchez Alvarado, 2013). For example, after irradiation ablates neoblasts and depletes their recent division progeny, planarians are incapable of regenerating new tissues. Yet, the surviving differentiated cells still display normal transcriptional responses after amputation. In the complete absence of neoblasts, differentiated cells upregulate expression of early wound-response genes, in addition to re-specifying the A/P axis within 1-day post-amputation (Gurley et al., 2010; Ogawa et al., 2002; Petersen and Reddien, 2009; Reddien et al., 2007; Tasaki et al., 2011). It remains to be determined whether a niche for neoblasts exists. But it is unlikely that stem cells and their local microenvironment will be the only elements that will need to be deciphered in order to understand animal regeneration. Something about the nature of the global pre-existing differentiated tissues could be an important factor in determining to what extent an animal—whether it be a planarian or a human—can regenerate.

To obtain a comprehensive picture of the cellular and tissue factors that may be enabling regeneration, we recently took advantage of single-cell sequencing technology to generate a comprehensive atlas of whole-body regeneration. By defining the expression profiles of nearly 300,000 cells at 7 different time points and under three different conditions, we defined comprehensive profiles of both successful and unsuccessful planarian regeneration. Analyses of these data identified the existence of previously unsuspected transient regeneration-activated cell states (TRACS) in the muscle, epidermis and intestine (Benham-Pyle et al., 2021). The identified TRACS appear at defined timepoints during regeneration and are undetectable once regeneration is completed. Importantly, genetic disruption of TRACS blocked regeneration (Fig. 3B), thus demonstrating that cells other than stem cells or their direct progenitors are indeed necessary for the coordination of regeneration across scales and possibly long distances.

The discovery of TRACS supports the notion that terminal differentiation, while possible in some instances, is not necessarily the end game of adult cells. Instead, it may be much more useful to think of the differentiated state of adult cells as merely being stable instead. There is, of course, still much to be learned about TRACS. Chiefly, understanding how they arise and how they are distributed across the animal body plan. Moreover, given their transient nature, we would benefit from understanding their fate. Do cells in TRACS differentiate? Do their return to their prior state or move on to different states? Once they exit the state are these cells reactivated by subsequent injuries? Future studies aimed at defining the mechanisms that underpin the function of TRACS in cell–cell communication should address this matter. We anticipate that this novel cellular state is likely taking place in other organisms and that its mechanistic dissection may ultimately help us better understand not only regeneration but also aid in designing strategies to drive regeneration in tissues and organs with poor regenerative abilities.

## 6. Concluding remarks

In my message as president to the Society for Developmental Biology in 2020 (Sánchez Alvarado, 2020), I wrote: “For most of the 20th century, modern developmental biology has been limited to the study of a handful of organisms in great part due to the absence of technology that prevented us from taking a more systematic and global approach to understanding fundamental aspects of developmental processes. That limitation is no longer as daunting. Why then should we continue to bring nature into our labs when it is becoming more and more practical to bring our laboratory sophistication to nature and study development there instead?” Imagine the immensity of what developmental biology can contribute in the in the next 100 years to address pressing global problems merely by expanding our interrogation of developmental processes into unknown and/or understudied organisms. Organisms with which we share profound evolutionary kinship. Simply put, we have but barely scratched the surface of development: **we do not know what is already possible**. The sheer number of species out there waiting to show us what is indeed biologically possible is staggering. Nature has done many more experiments than any of us can fathom, each extant species a unique interpretation of evolution. Equally remarkable is the fact that our species has the necessary tools to decode and understand them all if we so wished. In fact, merely expanding our knowledge of developmental processes in as many species as possible would stand to provide unimagined knowledge, which would result in “undreamed-of utility” (Flexner, 1939) and more than likely help discover new, unsuspected, and thus far undetected fundamental principles in biology.

## Figures and Tables

**Fig. 1.**
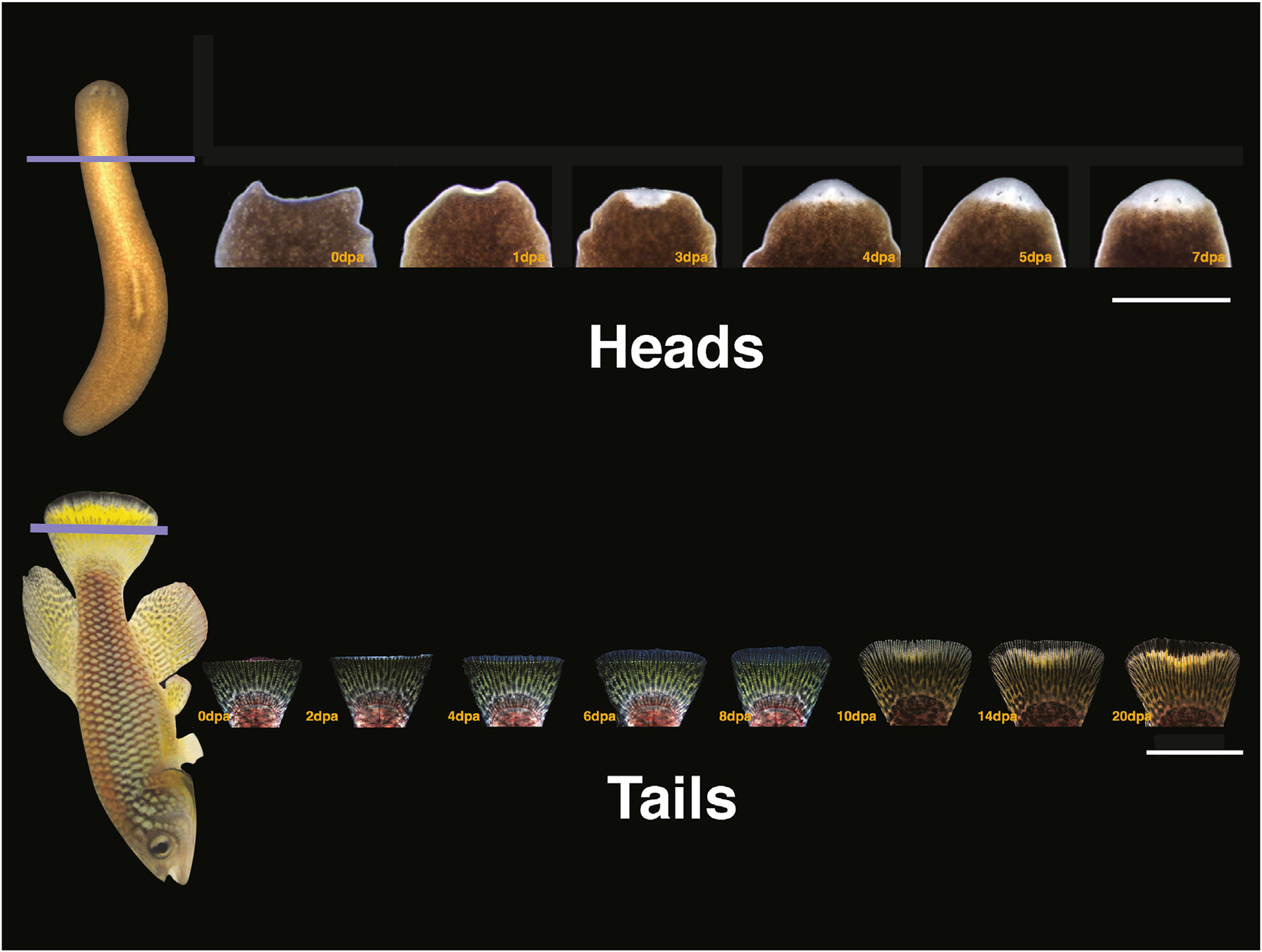
Examples of Invertebrate and vertebrate regeneration. Top panel shows a time series of head regeneration after amputation in planaria species *Schmidtea mediterranea*. Scale bar: 500 μm. Bottom panel shows a time series of tail (caudal fin) regeneration in the freshwater teleost fish *Nothobranchius furzeri* (Wang et al., 2020). Scale bar: 7 mm.
